# Acid hydrolysis of *Solanum tuberosum* periderm for accumulation of polyhydroxyalkanoates in *Pseudomonas putida* MTCC 2475

**DOI:** 10.3389/fbioe.2024.1343540

**Published:** 2024-02-07

**Authors:** Sonika Kag, Pravir Kumar, Rashmi Kataria

**Affiliations:** ^1^ Department of Biotechnology, Delhi Technological University (DTU), Delhi, India; ^2^ School of Bio Sciences and Technology, Vellore Institute of Technology (VIT), Vellore, Tamil Nadu, India

**Keywords:** acid hydrolysis, fermentation, *Solanum tuberosum* peiderm, *Pseudomonas putida*, polyhydroxyalkanoate, potato peel waste

## Abstract

Polyhydroxyalkanoates are a class of biodegradable, biocompatible polymers composed of polyesters of R-hydroxyalkanoic acids and deposited intracellularly by a variety of microorganisms which have potential to serve as alternative to commercial plastic. Bioplastics are gaining attention due to sustainability, biodegradability, biocompatibility, and lower carbon footprint. Nevertheless, the commercialization of PHA is predominantly hindered by the elevated production expenses arising primarily from the use of a pure sugar substrate. Our study has established a feasible method for bioplastic formation applying *Pseudomonas putida* MTCC 2475 and *Solanum tuberosum* periderm as a carbon source. To optimize the sugar yield response surface methodology was used, which released 69.34% ± 0.25% reducing sugar. PHA production experiments were performed in hydrolysate containing media as well as commercial sugar containing mineral salt media. After 48 h of fermentation of using this sugar, a biomass concentration of 2.19 gL^−1^, with a PHA production of 0.60 gL^−1^ (28.71% ± 0.55%) was obtained which was comparatively similar with synthetic media (2.56 gL^−1^ cell dry weight and 29.97% ± 0.45% PHA). Furthermore, the monomers of PHA produced by hydrolysate were characterized using Gas chromatography-mass spectrometry, Fourier transform infrared spectroscopy, differential scanning calorimetry, and nuclear magnetic resonance. This investigation has identified three distinct monomers of medium-chain PHAs, namely, methyl 3-Hydroxydodecanoate, 3-Hydroxytetradecanoate, and Hexadecanoic acid 3-Hydroxy methyl esters. Hence this study concludes a sustainable production of bioplastics from *S. tuberosum* periderm waste.

## 1 Introduction

According to “Food and Agricultural Organization of the United Nations,” globally, annual *Solanum tuberosum* or potato production exceeds 40 million tons, resulting in substantial quantities of *S. tuberosum* waste. Approximately 40% of which is generated as byproducts in the food processing industry, including products such as fries, chips, and other packaged food (Rodríguez-Martínez et al., 2023). Commonly *S. tuberosum* periderm waste (potato peel waste) is utilized for generating low-value animal fodder, compost, or raw substrate for biogas production, resulting in the squandering of valuable nutritional resources within it. *S. tuberosum* periderm possess antioxidant, antibacterial, and anti-inflammatory and further more properties (Khanal et al., 2023). However, the fundamental principle of the current study remains the efficient disposal of waste with the economic viability of processing advancement. In various studies *S. tuberosum* periderm was utilized for animal feed but proper treatment is required before subjecting it as a fodder for non-ruminants as it contains high amount of fibres to be digested (Akter et al., 2023). Despite lacking economic value, *S. tuberosum* periderm waste holds promise due to its significant carbohydrate content, making it an attractive feedstock to produce valuable compounds (Almeida et al., 2023).

The ongoing quest for sustainable and biodegradable alternatives to non-renewable, petrochemical plastics has been a driving force in scientific research within the field of biobased plastics, particularly polyhydroxyalkanoates (PHA). PHAs are intracellularly decomposable polyesters that various types of microbes accumulate as reserve food materials under stressful conditions (Wang et al., 2023). PHA is categorized based on its carbon chain length, with two main categories: short-length (3–5) and medium-length (6–12). Both types exhibit biodegradable properties, but medium-chain-length PHA (mcl PHA) holds significant industrial value due to its elastomeric and thermoplastic characteristics (Zhou et al., 2023). Large-scale production of Polyhydroxyalkanoates has become possible by selecting highly potent microbial strains to ferment refined feedstocks (de Souza Reis et al., 2020). While fatty acids and sugars are commonly used carbon sources for PHA production. Hence, carbon-rich waste materials can serve as cost-effective, sustainable, and economical substrates for industrial-scale PHA production (Kacanski et al., 2023).

*S. tuberosum* biomass contains 40%–80% sugar/gram of dry weight in the form of starch, cellulose, and hemicellulose. Worldwide production of *S. tuberosum* is consistently on the rise, reaching approximately around 390 million tons in the year 2021 and its industrial processing generates large volume nearly 78–195 million tons (20%–50%) raw material (Remedios and Domingues, 2023). It has been explored to produce various renewable metabolites, including bioethanol (Rodríguez-Martínez et al., 2023), biobutanol (Abedini et al., 2020), lactic acid (Liang et al., 2014), and bacterial cellulose (Abdelraof et al., 2019). Additionally, research has demonstrated the potential for bioplastic production from potato peel starch, both through chemical methods (Bezirhan Arikan and Bilgen, 2019), and through microbial routes, such as SCL polymer production using *Bacillus megaterium* (Vu et al., 2021), and mcl PHA production using various strains of *Pseudomonas* (Tanikkul et al., 2020a; Mahato et al., 2021; Pan et al., 2021).

To the best of author’s knowledge, this study marks the first report of polyhydroxyalkanoate (mcl PHA) production using *S. tuberosum* periderm sugar as a carbon source by *Pseudomonas putida MTCC* 2475. The research was designed to maximize the extraction of reducing sugar by optimizing sugar concentration through response surface methodology, followed by bacterial fermentation for PHA production. Furthermore, the study explores the capacity of *Pseudomonas putida* (MTCC 2475) to produce PHA in various growth media and over different time frames. Additionally, the produced PHA was characterized using FTIR, DSC, and NMR.

## 2 Material and methods

### 2.1 Sample collection

**FIGURE 1:**
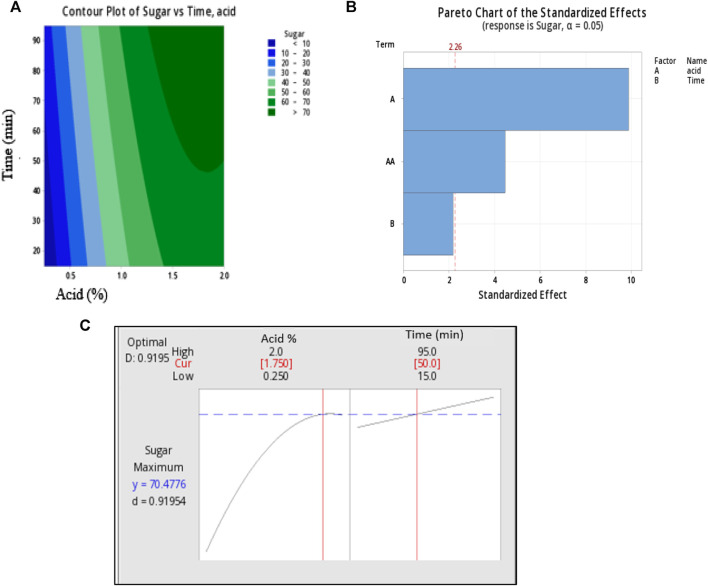
Response surface plots of two independence factors acid (X_1_) Acid concentration and Time (X_2_) where **(A)** is pareto chart of standardized effect, **(B)** counter plots and **(C)** optimum condition parameters.

*S. tuberosum* periderm biomass was obtained from the canteen of Delhi Technological University (DTU) (Figure 1). The processing involved washing the periderm and subsequently drying them at 40°C. A constant weight was achieved using Matrix Scientific Instrument. To achieve biomass size reduction in the range of 0.5–1 mm, a physical pretreatment involving grinding was applied.

### 2.2 Microorganism procurement and maintenance

The bacterium used in this research, *Pseudomonas putida* 2475, was procured from the Microbial Type Culture Collection (MTCC) in Chandigarh, India. Upon procurement, a mother culture was established on nutrient agar medium composed of the following components per liter: beef extract (0.1 g), yeast extract (2 g), peptone (5 g), NaCl (5 g), and agar (15 g), The pH of the medium maintained to be 7.

### 2.3 Acid hydrolysis of *Solanum tuberosum* periderm biomass

*S. tuberosum* periderm biomass is composed of various components, including starch, cellulose, lignin, protein, cellulose, and hemicellulose. To release fermentable sugars from these polysaccharides, an appropriate treatment strategy is necessary before subjecting the biomass to fermentation. Thermochemical treatment involving dilute acid at high temperatures is employed to convert the carbohydrates into monomer units and enhance sugar yield. For the optimization of sugar yield, a chemical treatment was carried out using varying HCl concentrations (0.25, 1.15, and 2%), and times (15, 55, and 95 min) at autoclave condition (121°C/15psi). For acid hydrolysis stock solution of 10% HCl (3.2 M) was prepared and then working solutions of 0.25%, 1.15%, and 2% of acid concentrations were prepared from it. Reaction volume was taken 1L and biomass loading was 100 g in 1,000 mL of aqueous phase in every single combination. After cooling centrifugation was done to separate liquid hydrolysate from unhydrolyzed solid part. After centrifugation liquid hydrolysate was subjected for detoxification with dry calcium carbonate until the pH of sample reaches up to neutral value (6.8–7).

#### 2.3.1 Design of experiment by response surface methodology for acid hydrolysis

To optimize the highest yield of reducing sugar, statistical software (Minitab) was employed for regression analysis. Thirteen successive trials were outlined using a Central Composite Design, involving two factors: acid concentration [0.2, 1.125% and 2% (w/v)] and time (15, 55, 95 min), with five central points for the single response variable (reducing sugar yield). Each of the 13 trials was conducted in triplicate with 10% biomass loading, and the standard deviation was recorded.

A regression equation was utilized to summarize the combined results of the two independent variables: acid percentage (**X**_**1**_) and time in minutes (**X**_**2**_). The equation, represented as Y (the response, expressed as a percentage of reducing sugar), was used to interpret the observed outcomes.

To account for unexpected variations in the acquired data, the trials were randomized. Model fitness, the **R** ^**2**^ value, and the F-test results were employed to assess the model’s effectiveness, using data from ANOVA. The interaction between the two independent variables was examined through counterplots and a Pareto chart. Model efficacy was evaluated by comparing experimental results with the model’s predicted values.

### 2.4 Composition analysis of raw *Solanum tuberosum* periderm biomass

The chemical composition of *S. tuberosum* periderm, including proximate analysis (total solid, moisture, and ash), was determined using techniques developed by the National Renewable Energy Laboratory (NREL) (Fatmawati et al., 2023). Briefly for total solid analysis 1 g biomass was kept in oven at 105°C till constant weight achieved. For the ash content analysis, the sample was subjected to a temperature of 575°C in a muffle furnace for a duration of 6 hours. Subsequently, the crucibles were taken out directly from the furnace and transferred into a desiccator, where they were allowed to cool for a designated period. Following this, the crucibles and ash were weighed and analyzed gravimetrically using prescribed formula % Ash=weight crucible plus ash−weight crucible÷Oven dry weight sample×100 .

Starch and total phenolics were quantified spectroscopically, following methods found in the literature (Lafont-Mendoza et al., 2018). In the starch analysis method 1 mL sample was taken in a test tube and 0.33 mL of potassium iodide (KI) solution was subsequently added. The blue color intensity was measured at 600 nm using spectrophotometer against KI blank. The amount of starch in the sample was determined by the standard curve (1 mg/mL). For total phenolics 1 gm sample was subjected to methanol extraction (1:10) and then subjected to incubation with Folin reagent (0.5 mL) and sodium carbonate (1 mL) for 2 h at room temperature and then subjected to spectrophotometric assay at 765 nm against the gallic acid standard (1 mg/mL) (Samarin et al., 2012). Cellulose and hemicellulose content was estimated using the chlorite method. Briefly holocellulose content was determined using treatment with sodium chlorite. 17.5% NaOH was used to extract the cellulose compound from the holocellulose. Hemicellulose was determined by subtracting the value of cellulose from holocellulose (Zhou et al., 2015).

### 2.5 Total reducing sugar estimation

After dilute acid treatment, Hydrolysate was used for reducing sugar analysis by the Dinitro salicylic acid method briefly 1 mL of extracted hydrolysate with equal amount of DNS solution (3,5 dinitro salicylic acid 1 gm, Na_2_SO_3_ 0.05 g, sodium potassium tartarate 18.2 g, NaOH 1 gm, and phenol 0.2 gm per 100 mL of H_2_O) were subjected to heating for 10 min at 100°C and optical density at 540 nm was recorded upon cooling using standard glucose curve (1 mg/mL). Total reducing sugars (glucose, fructose, and xylose) present in the biomass has been considered for estimation (Shangdiar et al., 2023).

$$Sugar\;recovered\;\left(\frac{mg}{mL}\right)=Volume\;ofliquid\;hydrolysate\;recovered÷Amount\;of\;sample\;taken\;for\;hydrolysis\times 100$$

### 2.6 Physicochemical characterization of *S. tuberosum* periderm biomass

Functional group analysis of both raw and acid hydrolyzed *S. tuberosum* periderm waste was conducted using Fourier-transform infrared (FTIR) spectroscopy with KBr pelleting (PEIR SUBTECH SPECTRUM ASCII PEDS 4.00). Thermogravimetric analysis (TGA) of the *S. tuberosum* periderm was carried out to observe decomposition peaks at various heating rates under an inert nitrogen environment, using a PerkinElmer analyzer. The analysis covered temperatures ranging from 30°C to 900°C with a heating rate of 10°C min⁻^1^. The surface structure of both raw and treated *S. tuberosum* periderm biomass was observed using a Scanning Electron Microscope (ZEISS EVO 18) at a beam accelerating voltage of 20 kV.

### 2.7 PHA production using *S. tuberosum* periderm biomass hydrolysate by *P. putida*

*Pseudomonas putida* (strain 2475) was procured from MTCC, Chandigarh, India. The culture was periodically revived by transferring it to fresh Luria Bertani media (comprising 5 g/L yeast extract, 10 g/L NaCl, 15 g/L agar, and 10 g/L tryptone) and storing it at a low temperature of 4°C. To develop the inoculum, bacterial colonies from the pure culture were transferred to 150 mL of nutrient broth and incubated at 30°C with continuous shaking at 180 rpm for 24 h. Initially, we assessed the growth pattern of *P. putida* by measuring its optical density at 600 nm using a GENESYS 50 UV-visible spectrophotometer from Thermo Fisher. This assessment was conducted in mineral salt media containing Na_2_HPO_4_·7H_2_O (30 g/L), KH_2_PO_4_ (15 g/L), NaCl (5 g/L), NH_4_Cl (1 g/L), MgSO_4_ (2 g/L), CaCl_2_ (0.1 g/L), and synthetic glucose (10 g/L), as the carbon source. For nitrogen limitation, the quantity of NH_4_Cl was reduced to 1 g/L. In media containing hydrolysates, 0.1% tryptone was used as nitrogen limitation.

For inoculation, 1% of the freshly prepared seed culture, prepared in Luria Bertani media with an optical density of 0.8 and a concentration of 312 × 10^6^ colony-forming units (cfu) was employed. The study involved three different media conditions for PHA production, each conducted in 2 L conical flasks containing 1 L of modified medium: **(A)** Modified medium comprised a specified volume (1%) of acid treated liquid hydrolyzed *S. tuberosum* periderm hydrolysate at a concentration of 10 g/L (after neutralization with calcium carbonate), 5 g/L NaCl, and 1 g/L tryptone. Incubation was carried out in a shaking incubator (180 rpm) at 30°C for 24, 36, 48, and 72 h. **(B)** Pure hydrolysate medium at a concentration of 10 g/L. **(C)** Mineral salt medium, as described in the literature (Sriyapai et al., 2022). PHA production values were averaged after each experiment, which was conducted in duplicate.

### 2.8 Cell dry weight measurement and extraction of PHA

After incubation, the culture broth was subjected to centrifugation at 10,000 rpm using an Eppendorf 5810 centrifuge for 15 min at 4°C. Following centrifugation, the supernatant was carefully drained, and the cell pellet was recovered. Recovered cell pellet was subsequently washed sequentially with 25 mL deionized water, 25 mL acetone, and 25 mL ethanol, and then dried at 40°C until a constant weight was achieved. This recovered cell biomass, referred to as cell dry mass, was determined gravimetrically, as per the method outlined in the literature (Tanikkul et al., 2020b). Polymer extraction was carried out using the chloroform extraction method with slight modifications, following the procedure described by Filippi et al. (2021). 2.1 g cell pellet was crushed with the help of mortar pestle and then incubated in 20-fold chloroform (43.8 mL) at 60°C in a water bath for 2 h. The resulting mixture was filtered using Whatman filter paper (1), and the liquid was concentrated through evaporation. PHA was obtained by precipitation of whole recovered solution obtained after heating with 22 mL ice-chilled methanol and subsequently analyzed using GC-MS, following the previously established protocol (de Souza Reis et al., 2020). Briefly For the analysis of monomer composition, we performed GC MS-MS. First, 20 mg of dry cell biomass was subjected to acidic methanolysis. Methyl esters were prepared by combining 15% sulfuric acid in 85% methanol (2 mL) with chloroform (2 mL) in a screw-capped culture tube and heating it at 100°C for 2 h and 20 min (de Souza Reis et al., 2020).

### 2.9 Monomer identification GCMS-MS analysis

After cooling, 2 mL of water was added to separate the organic layer from the aqueous layer. The GC-MS-MS analysis was conducted using the Triple quadrupole 7000D GC/TQ Agilent, equipped with a triple-axis detector. For the analysis, PHA was used in the form of hydroxy alkanoic acid methyl esters, following the method reported by Mahato et al. (2021). A sample of 2 μL at a split ratio of 1:50 was automatically injected into the GC and injection temperature was 200°C. Helium served as the carrier gas at 48 mL^−1^min and 0.42 bar pressure (Hierro-iglesias et al., 2023). Monomer identification was performed using the NIST 17 library.

### 2.10 Preparation and characterization of PHA film

The extracted PHA film was prepared in a fume hood by dissolving 200 mg extracted PHA in 20 mL of chloroform at room temperature and subsequently pouring the solution into Petri dish till complete evaporation of solvent. To determine the melting temperature of the PHA film, we conducted Differential Scanning Calorimetry (DSC) using a temperature-regulated system (DSC 8000, Perkin Elmer). We took 5 mg of extracted PHA in an aluminum pan and subjected it to a nitrogen flux rate of 10 mL/min, heating it from 30°C to 250°C, following the procedure outlined in the literature (Kacanski et al., 2023) with some modifications.

For the analysis of functional groups, we recorded Infrared (IR) spectra in the range of 4,000 to 450 cm^−1^. This was done by preparing KBr pellets and using a (Perkin Elmer Frontier Shelton CT08484). In brief potassium bromide (KBr) was subjected to drying at 110°C for 2 h for moisture removal. 5 mg sample was mixed with 200 mg of powdered KBr. After pulverization, mixture (sample and KBr) placed in to pellet forming machine and resulting pellet was taken for IR analysis (Cerrone et al., 2023). To analyze the chemical shift of the PHA, we utilized 1H Nuclear Magnetic Resonance (NMR) by dissolving a 5 mg sample in 600 μL of CDCl_3_, employing a (BRUKER Proton NMR 500 MHz), as described by Kacanski et al. (2023). The chemical shift of PHA was recorded in parts per million (ppm).

## 3 Results and discussion

### 3.1 Composition analysis

The composition analysis of *S. tuberosum* periderm collected during winter was conducted, and the findings are presented in Table 1. One gram of the sample was used for composition analysis, and the results were reported in triplicate.

**TABLE 1:** Composition analysis of *S. tuberosum* periderm waste.

Parameters	% (W/W)
Total solid	90.366 ± 0.40
Moisture	09.633 ± 0.40
Ash	14.033 ± 0.05
Total lignin	06.564 ± 0.20
Starch	64.44 ± 0.26
Total phenolics	0.6 ± 0.09
Cellulose	9.01 ± 0.48
Hemicellulose	1.89 ± 0.48
Total solid	90.366 ± 0.40

The composition of the *S. tuberosum* periderm was as follows: starch 64.44%, ash 14%, total lignin 6.5%, phenolics at 0.6% of *S. tuberosum* periderm, cellulose 9.01%, hemicellulose 1.89%, and the total solid content was found to be 90% (w/w).

In comparison, Malakar et al. (2020) reported 76% moisture and 8% ash in S. tuberosum periderm, Arapoglou et al. (2010) reported starch content at 52.14%, Lima et al. (2021) reported cellulose and hemicellulose content ranging from 10% to 30%, and Samarin et al. (2012) reported a total phenolic content of 522 µg GAE/g dry weight. These findings support the results of our study. However, it is worth noting that the composition of *S. tuberosum* periderm can vary among different *Solanum tuberosum* species (Sampaio et al., 2020).

### 3.2 Acid hydrolysis of the biomass

Different concentrations of dilute acid and time variation for hydrolysate development were used in this study which resulted in a varied amount of reducing sugar and their combined severity factor (LogR_o_). Based on the above parameters, results presented in Table 2. From the obtained results it can be concluded that very low acid concentration (0.25), is unable to liberate high sugar at any time. Briefly 0.25% acid at 15 min extracted 0.05% reducing sugar. However, at 55 and 95 min sugar yield was 5.49% and 16.45%, respectively. Center point (1.12% acid and 55 min time) was obtained through the software and the sugar yield in all the five runs was between 59% and 63%. While High acid concentration (2%) releases the highest amount of sugar such as 76% at 15 min, and 70% at 95 min. From the above observations it can be said that sugar yield increases with the increase in acid concentration. However, time is not very significant factor in terms of sugar removal as 2% acid at 15 min can extract more sugar as compare to 95 min. The reason behind the diversity in sugar removal is high acid concentration and longtime combinedly generate toxic metabolites which leads to degrade sugar molecule. Therefore, a comparatively low acid concentration was optimized from response surface methodology the for maximum sugar with low toxicity. Further the optimized condition was used for acid hydrolysis.

**TABLE 2:** Experimental conditions, sugar recovery (%) and severity factor of acid hydrolysed *S. tuberosum* periderm by central composite design.

Run order	Time (X1)	Conc. (X2)	Sugar (%)	Combined severity factor
1	55	2	65.12 ± 0.17	0.18
2	95	0.25	16.45 ± 0.30	1.26
3	55	0.25	5.49 ± 0.16	1.52
4	15	0.25	0.05 ± 0.04	0.75
5	95	2	70.80 ± 0.27	1.83
6	55	1.125	61.39 ± 0.23	1.83
7	95	1.125	67.36 ± 0.41	1.83
8	55	1.125	60.99 ± 0.11	1.83
9	15	1.125	35.72 ± 0.26	1.83
10	55	1.125	61.58 ± 0.06	2.09
11	15	2	76.64 ± 0.17	0.99
12	55	1.125	59.82 ± 0.13	2.07
13	55	1.125	63.67 ± 0.28	2.33

X1 and X2 are the coded coefficients of time (min) and acid concentration (%), respectively. And combined severity factors LogR0 = Log [t· exp ((Hydrolysis temperature ˗ reference temperature)/14.75)]- PH where t represents time (min), the hydrolysis temperature (°C) and Tref the reference temperature, which is usually set to 100°C (Liang et al., 2014).

### 3.3 Optimization of hydrolysis condition through RSM

This study was designed to establish a linear equation based on thirteen experimental runs, including five center points, all subjected to analysis of variance (ANOVA). Two factors, time and acid concentration, were optimized through Central Composite Design (CCD) and further analyzed using regression analysis and analysis of variance. The independent variables and their respective values are presented in Table 3.

**TABLE 3:** *Solanum tuberosum* periderm experimental range of levels of independent process variables and coded values in CCD.

Variables	Level		
	−1	0	1
Time (min)	15	55	95
Acid concentration (%)	0.2	1.125	2

**FIGURE 2:**
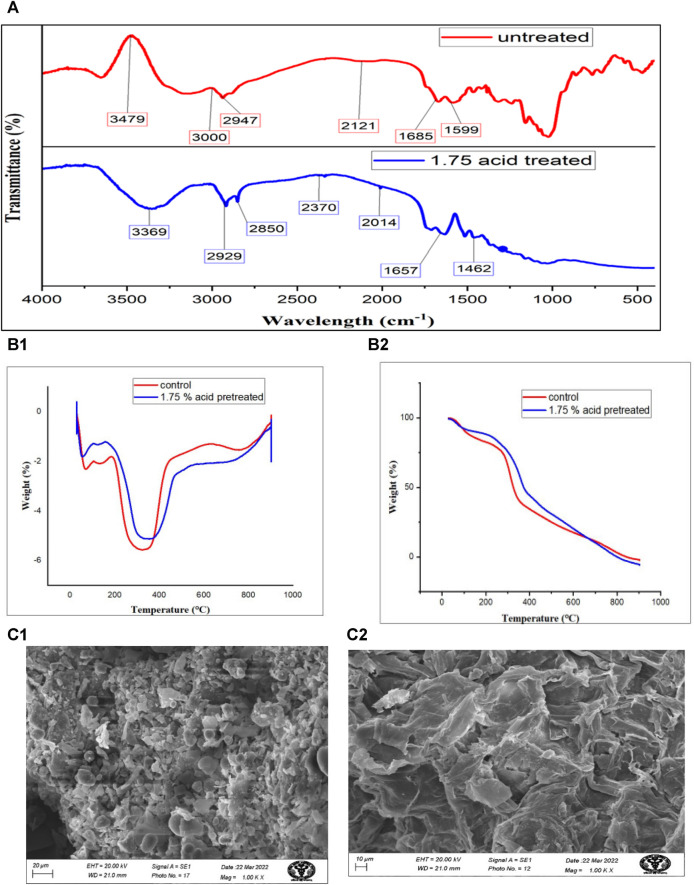
Thermochemical characterization of *S. tuberosum* periderm waste before and after acid hydrolysis **(A)** represents the FTIR of untreated and treated sample while, **(B1,B2)** belong to TGA and DGA, respectively and **(C1,C2)** are images obtained from scanning electron microscopy.

Following the completion of thirteen trial runs, the optimal conditions for achieving the highest yield of reducing sugars were determined using Minitab software and graphically represented in Figure 2. The Pareto chart illustrates the standardized effects of individual factors: (A) acid concentration, (B) time, and the combined effects of both factors. The Pareto chart indicates that factor (A) exerts the most significant influence on sugar extraction, while the impact of time is relatively negligible. Furthermore, the combined influence of acid and time explains a smaller portion of sugar extraction.

The contour plot displays the region of maximum sugar yield, falling within the ranges of 1.5%–2% for acid concentration and 50–90 min for time. Based on above observations, this study was aimed to exact the maximum sugar at mild acid concentration. At 2% acid concentration and after 95 min of hydrolysis time, sugar was observed to be 70.80 ± 0.27, however, the acid concentration and time was a bit higher. While at 2% acid and 15 min of time sugar yield was maximum (76%) but acid concentration was higher. Hence, the maximum sugar liberation and milder condition were established to obtain optimized condition. The predicted optimal conditions from the model were determined for 1.75% acid concentration and 50 min of reaction time, resulting in a predicted sugar yield of 70.47%. To validate the accuracy of the predicted response, a hydrolysis experiment was conducted under the specified parameters, and the trials were conducted in triplicate. The average sugar yield obtained was 69.34% ± 0.25%, which closely matched the predicted value. Further this extracted sugar used as a carbon source for PHA production by *P. putida* in through this study.

The combined severity factor (LogR_o_) under the optimized conditions was calculated to be 1.99. In a study, 5% hydrochloric acid, yielding 62 g L^−1^ of sugars (Khanal et al., 2023). In a two stage hydrolysis study, 32 g of potato peel in 400 mL of 1% w/w H_2_SO_4_ at 180°C for 60 min was performed, For second stage hydrolysis with cellulase, α-amylase and glucoamylase 36 g L^−1^ glucose was obtained (Abedini et al., 2020). In another two-step hydrolysis process of potato peels, 141 g L^−1^ of sugar obtained with 3% w/w H_2_SO_4_ in an autoclave condition for 15 min, The second stage enzymatic hydrolysis was done with the crude enzyme complex produced by *Aspergillus* sp. For 72 h at 50°C and 150 rpm (Soni et al., 2023). In an experiment, potato peel waste underwent pretreatment with 1% H_2_SO_4_ at 121°C for 30 min, with a substrate loading of 10%. In the subsequent hydrolysis step, 10 g of the pretreated substrate were exposed to enzymatic hydrolysis using cellulase, hemicellulose and amylase (at concentration of 30 U, 5 U, and 70 U/g of substrate, respectively). This hydrolysis process occurred at 50°C in a rotatory shaker incubator, with agitation at 700 rpm for 48 h. The outcome of this process revealed the presence of 77 g/L of reducing sugar (Kalafat et al., 2018).

In the current study, we achieved a considerably high yield of reducing sugars at 1.75% HCl, which is milder in terms of the generation of toxic byproducts such as furfural and hydroxymethylfurfural. Details regarding the responses after the trial runs and their Coefficient of Standard Deviation (CSD) are provided in Table 2.

RSM (Response Surface Methodology) was employed to assess the combined impact of two independent variables: Acid concentration **(X**_**1**_ **)** and Time **(X**_**2**_ **)**, From the numerical data obtained in this study, the simplified linear regression model for sugar yield from S. tuberosum periderm can be expressed as follow

$$‐24.19+93.8X_{1}+0.1758\;X_{2}\;–\;25.54\;X_{1}\times X_{1}$$

where **X**_**1**_ = Acid concentration (%) and **X**_**2**_ = Time (min).

The regression analysis of the trial runs yielded a statistically significant result, with an R-squared value of 93.14%, indicating a strong fit of the model. Additionally, the lack-of-fit for the model was found to be negligible. The output of the regression analysis for glucose yield is presented in Table 2, where a *p*-value greater than 0.05 indicates the significance of the model.

To create the best-fit model and eliminate irrelevant conditions (those with *p*-values greater than 0.05), a linear equation for regression was applied. So, a model for the optimization of reducing sugar yield from *S. tuberosum* periderm was successfully generated. Based on obtained values, the sequence of relevance for the two factors on acidic hydrolysis was acid concentration > time.

The counterplots and Pareto chart were used to visually depict the effects of individual variables and the combined interactions of acid concentration (X_1_) and Time (X_2_) on sugar yield.

These statistical analyses and graphical representations are valuable tools for understanding the impact of the variables on the hydrolysis process and optimizing the yield of reducing sugars from *S. tuberosum* periderm waste.

### 3.4 Thermochemical characterization of biomass

#### 3.4.1 FTIR

**FIGURE 3:**
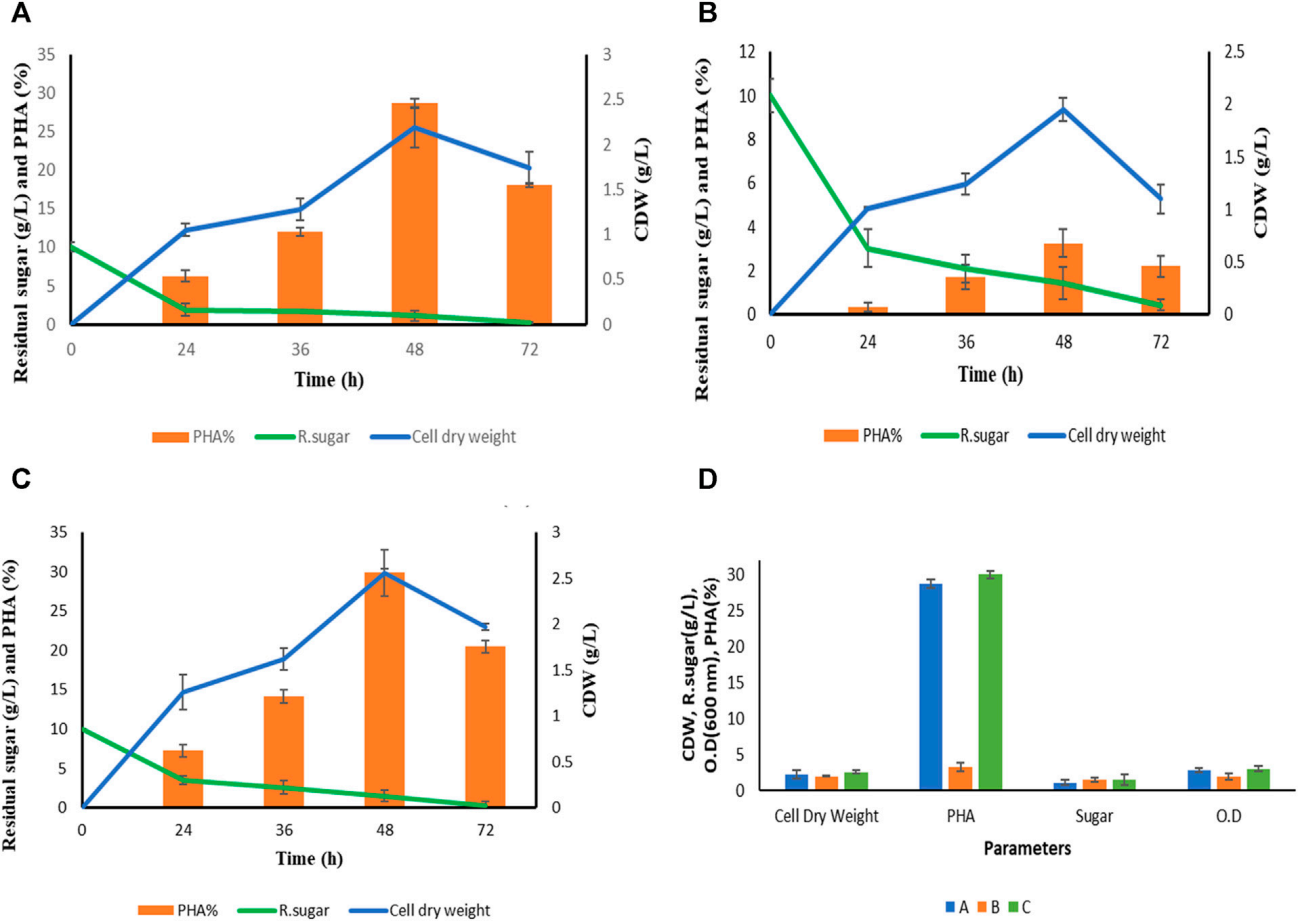
Summary of growth pattern of *P. putida* in terms of optical density (OD), cell dry weight (cdw) and residual sugar in three different media. In media **(A)** This includes extracted hydrolysate from *S. tuberosum* periderm, NaCl, and tryptone. In media **(B)** This involve pure hydrolysate at a concentration of 10 g L^−1^ without the addition of any other components. In media **(C)** Mineral salt media containing Na_2_HPO_4_.7H_2_O, KH_2_PO_4_, NaCl, NH_4_Cl, MgSO_4_, CaCl_2_ and Synthetic glucose. **(D)** Growth pattern of *P. putida* in terms of cell dry weight, PHA, residual sugar and optical density at optimized condition (48 h).

Infrared (IR) studies were conducted to assess the chemical modifications in *S. tuberosum* periderm waste before and after acid hydrolysis treatment. Key peaks in the FTIR spectra of treated and untreated biomass are depicted in Figure 3A. Treated samples exhibited lower peak intensities compared to untreated ones, indicating the efficient removal of various biomass constituents.

In the untreated sample, a peak at 3,480 cm^−1^, corresponding to the free and intermolecular O-H stretch, suggests the presence of starch. However, in the case of treated biomass, a reduction in peak intensity and a shift in the band to 3,369 cm^−1^ indicate the conversion of starch into glucose.

In the untreated sample, the peak at 3,000 cm^−1^ corresponds to the free hydrogen-bonded OH stretching of cellulose. The band at 2,947 cm^−1^ represents the stretching of functional groups, methyl and methylene of cellulose. Post-treatment, there is a significant reduction in cellulose content, as evidenced by the low-intensity peak at 2,850 cm^−1^ as compared to untreated sample (3,000 cm^−1^)

Sharp peaks observed between 1,657 and 1,463 cm^−1^ in treated *S. tuberosum* periderm are attributed to the C=C stretching of the aromatic structure of suberin. This peak distribution pattern is consistent with findings from previous studies, such as the work of Malakar et al. (2020) on acid-treated *S. tuberosum* periderm waste and the research conducted by Liang and McDonald (2014), which compared raw biomass with its residue after fermentation. Both studies reported similar patterns in O-H stretching, C-H (indicative of carbohydrate presence), C=O, and C-O-C (related to hydroxy fatty acid and suberin).

#### 3.4.2 TGA

Acid hydrolyzed (1.75%) and unhydrolyzed *S. tuberosum* periderm underwent thermogravimetric analysis to compare their thermal decomposition rates, as illustrated in Figure 3B. The Differential Thermogravimetric (DTG) analysis of peel biomass revealed two distinct weight loss regions in all samples.

The first weight loss peak, observed at 100°C, can be attributed to dewatering processes. Weight loss occurring between 200°C–300°C indicates the depolymerization of cellulose and hemicellulose, key components of the biomass. Additionally, a peak in the temperature range of 350°C–380°C corresponds to the cleavage of glycosidic bonds within starch molecules.

In the case of unhydrolyzed (raw) samples, the TGA (Thermogravimetric Analysis) curve exhibited a peak at 354°C, resulting in a 59.941% weight loss. However, under optimized conditions of 1.75% acid concentration and approximately 50 min of treatment, a more significant weight loss of approximately 60% was observed at 429°C. This peak signifies the effective removal of starch from *S. tuberosum* periderm due to acidic hydrolysis.

The thermal decomposition behavior, as assessed through TGA and DTG, in our *S. tuberosum* periderm samples exhibited similarities with the findings of Liang et al. (2014) Notably, distinct peak areas corresponding to starch, cellulose, and hemicellulose (within the range of 220°C–400°C) were evident. In contrast, lignin and suberin displayed notable peaks at temperatures between 250°C–290°C.

Investigating the thermal properties of *S. tuberosum* periderm through Thermogravimetric Analysis (TGA) revealed noteworthy findings. The TGA analysis demonstrated distinct decomposition peaks for cellulose and hemicellulose in the temperature range of 200°C–375°C, while lignin exhibited a degradation peak spanning from 180°C to 450°C, consistent with the observations made by Osman et al. (2019). In a separate study conducted by Liang et al. (2015), bio-oil was produced from *S. tuberosum* periderm, and it was observed that the thermal degradation of the material occurred at temperatures below 450°C. These findings further corroborate the presence of these specific biomolecules within the *S. tuberosum* periderm sample. This alignment between the identified peaks and their corresponding thermal degradation patterns is consistent with the existing literature.

#### 3.4.3 SEM

The surface morphology of both pre-treated and untreated *S. tuberosum* periderm waste was analyzed using scanning electron microscopy (SEM), as depicted in Figure 3C. The images reveal distinct disparities between the pre-treated and untreated samples. In contrast to the untreated sample, which appears compact and smooth, the pre-treated sample exhibits noticeable structural alterations.

These structural changes can be attributed to the acidic treatment, which appears to have resulted in the breakdown of bonds between lignin and carbohydrates. This structural modification likely played a role in facilitating the release of maximum sugars from the *S. tuberosum* periderm waste. In a recent study conducted (Soni et al., 2023), the authors examined both untreated and pretreated *S. tuberosum* periderm samples using scanning electron microscopy (SEM). Their observations closely parallel the findings of our current study. In their investigation, the untreated *S. tuberosum* periderm samples displayed an intact and solid structure. In contrast, the acid-pretreated samples, treated with 3% H_2_SO_4_, exhibited noticeable changes, characterized by cracking and fragmentation of the biomass.

### 3.5 Growth of *Pseudomonas putida* for PHA production and it is extraction in different production media

#### 3.5.1 Growth pattern of *Pseudomonas putida* in different media

**FIGURE 4:**
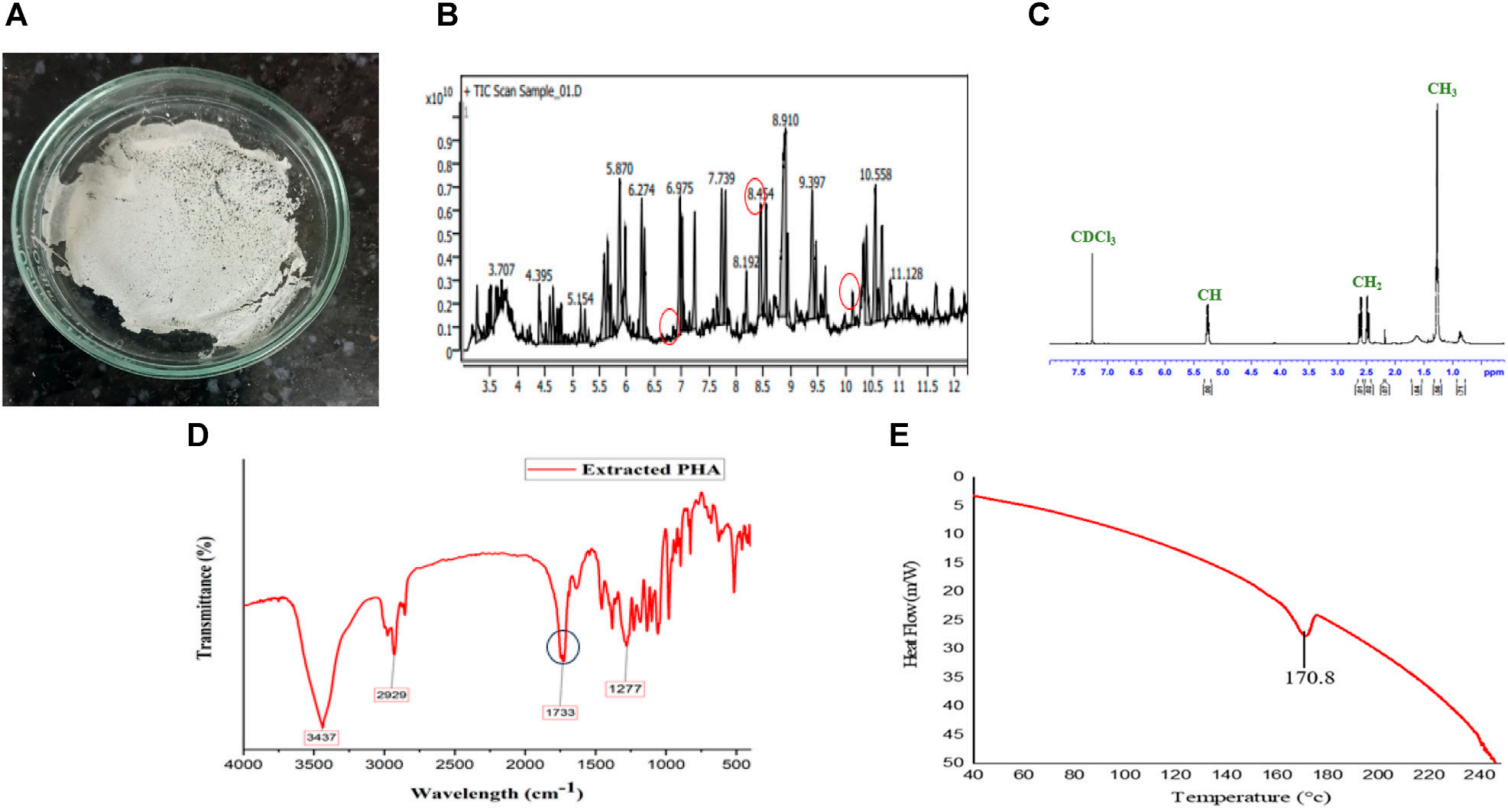
Extracted PHA characterization, Figure depicts **(A)** PHA film, **(B)** monomer confirmation of MCL PHA by GCMS-MS, **(C)** NMR spectra, **(D)** FTIR, and **(E)** DSC of PHA extracted from *Pseudomonas putida*.

The growth pattern of *Pseudomonas putida* was monitored in various production media denoted as A, B, and C as illustrated in Figure 4. Cell biomass was collected at distinct time intervals: 24, 36, 48, and 72 h, across the four different media mentioned above. An interesting trend emerged where biopolymer accumulation within the bacterial cells decreased after 48 h of fermentation in all media. This reduction can be attributed to the depletion of the carbon source. Notably, deposited Polyhydroxyalkanoates (PHA) within the cells serve as an energy source, allowing the cells to persist in carbon-depleted conditions, as discussed by Maheshwari et al. (2018). The highest Cell Dry Weight (CDW) was recorded at the 48-h mark in each production medium.

Figure 4 provides comprehensive data on the type of media, time (in hours), cell dry weight (in grams), PHA content (%), and residual sugar levels after each fermentation experiment. Mineral salt media containing synthetic glucose emerged as the most efficient PHA accumulator, with a PHA content of 29.97% ± 0.45%. This is attributed to the presence of the desired salt concentration, which optimally supports the growth of *P. putida*. However, it is important to note that the primary objective of this study is to utilize a sustainable carbon source, specifically *S. tuberosum* periderm, to reduce the production cost of PHA. Carbon source costs typically account for approximately 50% of the overall production expenses, as discussed by Jiang et al. (2016).

Hence, mineral salt media **(C)** was included primarily for comparative purposes, as it contains synthetic glucose, while the focus remains on evaluating *S. tuberosum* periderm hydrolysate (extracted sugar) as a potential carbon source for PHA production. Comparing the media types, it is observed that aside from mineral salt media, cell dry weight was slightly higher in the modified media **(A)**, which includes *S. tuberosum* periderm hydrolysate, tryptone, and NaCl, as compared to media (**B)** consisting solely of hydrolysate (1%). While **(D)** represents the O.D at 600 nm, cell dry weight, PHA, and residual sugar of all the three media (A, B, and C) at optimum condition (48 h).

Significant differences in PHA accumulation among these media types indicate that PHA-producing bacteria require a specific balance between nutritional stress and carbon abundance to foster PHA production. In contrast, when using only *S. tuberosum* periderm hydrolysate, while the carbon content was at the desired level (1%), the absence of NaCl to maintain osmotic balance and nitrogen led to biomass that lacked biopolyester components, specifically Polyhydroxyalkanoates (PHA), as reported by Passanha et al. (2014).

These findings underscore the importance of optimizing the carbon source, nutritional conditions, and osmotic balance to achieve efficient PHA production, particularly when utilizing sustainable carbon sources such as *S. tuberosum* periderm waste.

#### 3.5.2 PHA accumulation in *Pseudomonas putida* in different media

The percentage of extracted PHA is calculated gravimetrically in each media which is shown in Figure 4 but for GCMS MS analysis only media **(A)** However, PHA production from *S. tuberosum* periderm waste is reported for the first time in this study with 0.60 g/L (28.71% ± 0.55%) PHA production at 48 h with media **(A)** which is close to PHA accumulation in media **(C)** mineral salt media (29.97% ± 0.45%) media (A) contained hydrolysate (1%) along with tryptone (0.1%) and NaCl (0.5%) while media (B) confined pure hydrolysate without any other nutrients. 0.5 NaCl was added in media (A) for the osmotic balance of bacteria and the stress was given in the form of 0.1% organic nitrogen (tryptone). However, Nitrogen limited media triggers the bacteria to store excess carbon in the form of PHA but for bacterial growth and physiological activity small amount of nitrogen is required. Although complete omit of nitrogen may interfere with PHA accumulating activity of bacteria. Therefore, in media (B) no significant PHA accumulation observed. In case of media (C) 1% synthetic glucose along with other minerals and for nitrogen stress inorganic nitrogen (0.1% NH_4_Cl was added. Therefore, higher PHA accumulation was seen in synthetic media also. Although, it is assumed that acid-hydrolyzed sugar has its stress factors (phenolics) which are responsible for the accumulation of high percentage of PHA. Sharma et al. (2012) reported 22.6% mcl PHA of CDW from an isolated strain of *Pseudomonas putida* LS46 which is closely related to KT2440 (a potent PHA-producing recombinant strain) by using synthetic glucose (2%) as a carbon source.

In a study by Sikkema et al. (2023), three distinct strains of *Pseudomonas putida*, namely, NRRL B-14875, KT2440, and GN112, were employed as microbial cultures for the assimilation of fatty acids as the carbon source. The growth medium consisted of 20 mM ammonium sulfate, 50 mM potassium phosphate, 140 µM calcium carbonate, 2 µM copper sulfate, and 0.1 mL of a trace element solution. The incubation was carried out at 30°C, with constant agitation at 200 rpm, for a duration of 68 h. The investigation yielded significant results, with NRRL B-14875 producing 29% Polyhydroxyalkanoates (PHA), KT2440 contributing 34% PHA, and GN112 strain yielding the highest PHA content at 35%. In a literature 6 g/L MCL PHA was obtained using *Pseudomonas* putida KT2442. In first step bacterial was subjected to grow in organic fraction of municipal solid waste 1 L along with 5 g yest extract, 2 g glucose, 1 g KCl, (NH_4_) SO_4_ 1 g and trace element solution and fed batch fermentation and second step was ethanol was added as a substrate of PHA production (de Vrije et al., 2023).

This study shows a satisfactory amount of PHA production using only sugar hydrolysate (1%) NaCl (0.5%) and a negligible amount of tryptone (0.1%). At economic point of view, high amount of sugar (∼70%) can be extracted from *S. tuberosum* periderm biomass which is comparatively higher than other lignin and complex sugars containing organic waste. The comparative study with *P. putida* with other carbon sources is discussed in Table 4. However, extracted sugar % can vary in different species of *S. tuberosum* periderm and applied peeling method but considerable amount of starch is present in *S. tuberosum* periderm, could be used for biodegradable bioplastic (PHA) production which is discarded otherwise.

**TABLE 4:** Comparison of PHA production by different strains of *Pseudomonas putida* on the basis of fermentation parameters such as carbon source, incubation time, agitation speed and results were obtained in the form of cell dry mass, PHA% and monomer composition.

S. No	Bacteria used	Carbon source	Fermentation condition	Cell dry weight	PHA % in CDW	Monomer	Reference
1	*P. putida* KT2440	Glycerol (1%)	Incubation time 72 h at 28°C	2.6 g L−1	20.70%	Methyl 3-Hydroxyhexadecanoic acid, methyl 3-hydroxyoctadecanoic acid, methyl 3-hydroxydecanoic acid, methyl 3-hydroxydodecanoic acid, methyl 3-hydroxytetradecanoic acid	(Xu et al., 2021)
2	*P. putida* GO19	Terephthalic acid	E2 media containing 0.42% carbon source at 30°C and at 200 rpm	—	23%	3-hydroxyhexanoic acid, 3-hydroxyoctan-oic acid, 3-hydroxy-dodecanoic acid, 3-hydroxydodecenoic acid	(Kenny et al., 2008)
3	*P. putida* (MTCC 2475)	*Solanum tuberosum* periderm	Modified media containing 1% sugar hydrolysate at 30°C for 48 h	2.19	28.71% ± 0.55% (0.60 g/L)	Methyl 3-hydroxydodecanote methyl 3 hydroxytetradecanote hexadecanoic 3 hydroxy methyl esters	Present study

### 3.6 PHA monomer conformation

**FIGURE 5:**
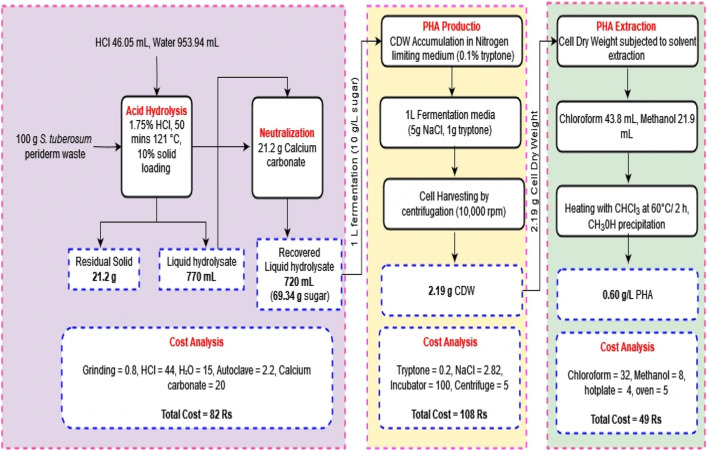
Mass balance study of PHA production from *S. tuberosum* periderm waste.

Based on the GC-MS-MS analysis, using *S. tuberosum* periderm waste hydrolysate as the sole carbon source resulted in the formation of medium-chain length polyhydroxyalkanoates (mcl PHA) containing three distinct monomers: 3-hydroxydodecanoate (3HDD) with a retention time (RT) of 6.99 min and a peak area of 1.75%., 3-hydroxydecanoate (3HD) with an RT of 10.34 min and a peak area of 1.72% and 3-hydroxytetradecanoate (3HTD) with an RT of 8.52 min and a peak area of 19.2% (Figure 5B).

These identified monomers demonstrate the feasibility of producing industrially important mcl PHA with carbon sources of low cost, such as *S. tuberosum* periderm hydrolysate. This approach enables the construction of mcl PHA with chain lengths ranging from 5 to 14 carbons, making it an economically attractive option.

This finding aligns with the work of Mahato et al. (2021), who reported the presence of beta-3-hydroxybutyric acid (7.41%), 3-hydroxytetradecanoic acid methyl esters, and 3-hydroxyhexadecanoate (5.03%) in PHA produced by *Pseudomonas aeruginosa*. However, it is important to note that their PHA production was observed in mineral salt media containing different oils as carbon sources, which may not be as sustainable and could potentially incur higher costs compared to the approach used in the present study. In a study Ciesielska et al. (2017) reported mcl PHA monomers such as 3- hydroxyhexadecanoate and 3- hydroxyoctadecanoate from *Pseudomonas putida* KT2440 using oleic acid as a carbon source.

By comparing PHA monomers produced by *Pseudomonas* from different studies it is evident that PHA extracted from *P. putida* MTCC 2475 (present study) is mcl PHA.

### 3.7 Preparation and characterization of extracted PHA film

The PHA film was created by adding extracted PHA in solvent (chloroform) and stored securely under aseptic conditions at room temperature (Figure 5A). The Differential Scanning Calorimetry (DSC) thermogram presented in Figure 5D reveals the melting temperature (T_m_) of the produced Polyhydroxyalkanoates (PHA), which was found to be approximately 170°C. This temperature closely aligns with the melting temperature of 3-hydroxydecanoic acid (166°C), as reported in the literature (Sharma et al., 2017).

The identification of functional groups within the extracted PHA was accomplished through Fourier Transform Infrared Spectroscopy (FTIR), as illustrated in Figure 5C. The spectral pattern of functional groups closely resembled findings from Mahato et al. (2021), who identified different forms of decanoic acid methyl esters, including Methyl 3-hydroxytetradecanoate and hexadecanoic acid methyl ester, from *Pseudomonas aeruginosa*. Notable absorption bands included: 1) A band at 3,437 cm^−1^, attributed to the O-H stretching of the hydroxyl group in PHA. 2) The band at 2,929 cm^−1^, assigned to the CH_2_ group. 3) An absorption band at 1,733 cm^−1^, indicating C=O stretching vibration, confirming the presence of an ester bond in the PHA monomer. 4) A band at 1,277 cm^−1^, associated with the asymmetric C–O–C stretching vibration.

Furthermore, the Proton Nuclear Magnetic Resonance (NMR) spectrum of the PHA film is depicted in Figure 5E. The complex multiple resonance bands between 1.26 and 1.33 ppm (peak no. 1) suggest the presence of methyl groups (–CH_3_) in the PHA film. Multiple resonance spectra observed between 2.17 and 2.60 ppm (peak no. 2) confirm the existence of methylene groups (–CH_2_). The band position ranging from 5.22 to 5.27 ppm (peak no. 3) indicates the presence of methane (–CH) in the PHA polymer. Peak no. 4 corresponds to the solvent CDCl_3_. These observed peaks align with prior studies on medium-chain length PHA produced by *Pseudomonas* strains, as reported by Gumel et al. (2014). Comparing the results of the present study with findings from various previous investigations, it can be confidently affirmed that the extracted polymer is indeed medium-chain length Polyhydroxyalkanoates (PHA).

## 4 Mass balance and economic analysis of the study

The experiment commenced with 100 g of *S. tuberosum* periderm waste used for sugar extraction, with a solid loading of 10%. This extraction process yielded 69.34% ± 0.25%, of sugar. Out of the total sugar obtained, 10 g were used for the fermentation process per liter, resulting in the production of 2.19 g of dry cell weight and 0.60 g/L PHA. Conducting an economic assessment revealed that the production of 1 L media with a concentration of 0.60 g/L of PHA incurred a cost of 239 Rs. In contrast, the acid hydrolysis method utilized a total of 100 g of biomass, yielding approximately 70 g of sugar. For the subsequent fermentation, only 10 g of this sugar were utilized, allowing the preparation of an additional 6 L fermentation media. Scaling down the acid hydrolysis process for a single liter production media has the potential to decrease cost. Moreover, the cost of PHA can not be compared with conventional plastic because along with packaging material it possesses biodegradable, sustainable, eco-friendly, medical application properties.

**FIGURE 6:**
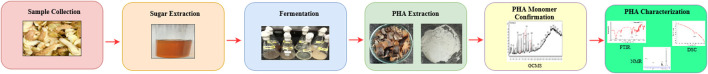
Steps involved in PHA production using *Solanum tuberosum* periderm.

Based on these results, it can be estimated that approximately 4.35 g of PHA can be produced from 100 g of *S. tuberosum* periderm waste. Figure 6 provides an operational flow diagram of mass balance and economic analysis, illustrating the process of PHA production from *S. tuberosum* periderm waste.

## 5 Conclusion

The data obtained from this study holds significant potential for enhancing the economic sustainability of Polyhydroxyalkanoates production from *S. tuberosum* periderm using *Pseudomonas putida*. The optimization of sugar concentration, achieved through RSM represents a critical step in PHA production, enabling higher sugar yields. While this study successfully demonstrated the production of MCL PHA 0.60 g/L within 48 h. The results encourage the concept of waste valorisation and its potentiality to donate to a more circular and sustainable environment. Although PHA production can be upgraded by optimizing certain parameters such as Carbon to nitrogen ratio and inoculum size. In summery the extent of the research extends further than the laboratory, motivating scientific community and industries to think about *Solanum tuberosum* periderm waste as a valuable feedstock in the endeavour of eco-friendly substitute to conventional plastic.

## Data Availability

The original contributions presented in the study are included in the article/supplementary material, further inquiries can be directed to the first and corresponding author.

## References

[B1] Abdelraof M. Hasanin M. S. El-Saied H. (2019). Ecofriendly green conversion of potato peel wastes to high productivity bacterial cellulose. Carbohydr. Polym. 211, 75–83. 10.1016/j.carbpol.2019.01.095 30824106

[B2] Abedini A. Amiri H. Karimi K. (2020). Efficient biobutanol production from potato peel wastes by separate and simultaneous inhibitors removal and pretreatment. Renew. Energy. Elsevier Ltd. 160, 269–277. 10.1016/j.renene.2020.06.112

[B3] Akter M. Anjum N. Roy F. Yasmin S. Sohany M. Mahomud M. S. (2023). Effect of drying methods on physicochemical, antioxidant and functional properties of potato peel flour and quality evaluation of potato peel composite cake. J. Agric. Food Res. 11, 100508. 10.1016/j.jafr.2023.100508

[B4] Almeida P. V. Gando-Ferreira L. M. Quina M. J. (2023). Biorefinery perspective for industrial potato peel management: technology readiness level and economic assessment. J. Environ. Chem. Eng. 11, 110049. 10.1016/j.jece.2023.110049

[B5] Arapoglou D. Varzakas T. Vlyssides A. Israilides C. (2010). Ethanol production from potato peel waste (PPW). Waste Manag. 30, 1898–1902. 10.1016/j.wasman.2010.04.017 20471817

[B6] Bezirhan Arikan E. Bilgen H. D. (2019). Production of bioplastic from potato peel waste and investigation of its biodegradability. Int. Adv. Res. Eng. J. 03, 93–97. 10.35860/iarej.420633

[B7] Cerrone F. Zhou B. Mouren A. Avérous L. Conroy S. Simpson J. C. (2023). Pseudomonas umsongensis GO16 as a platform for the *in vivo* synthesis of short and medium chain length polyhydroxyalkanoate blends. Bioresour. Technol. 387, 129668. 10.1016/j.biortech.2023.129668 37572888

[B8] Ciesielska J. M. Dabrowska D. Palasz A. S. Ciesielski S. (2017). Medium - chain - length polyhydroxyalkanoates synthesis by Pseudomonas putida KT2440 relA/spoT mutant: bioprocess characterization and transcriptome analysis. Amb. Express 7, 92. 10.1186/s13568-017-0396-z 28497290 PMC5427061

[B9] de Souza Reis G. A. Michels M. H. A. Fajardo G. L. Lamot I. de Best J. H. (2020). Optimization of green extraction and purification of PHA produced by mixed microbial cultures from sludge. WaterSwitzerl. 12, 1185. 10.3390/W12041185

[B10] de Vrije T. Nagtegaal R. M. Veloo R. M. Kappen F. H. J. de Wolf F. A. (2023). Medium chain length polyhydroxyalkanoate produced from ethanol by Pseudomonas putida grown in liquid obtained from acidogenic digestion of organic municipal solid waste. Bioresour. Technol. 375, 128825. 10.1016/j.biortech.2023.128825 36878376

[B11] Fatmawati A. Nurtono T. Widjaja A. (2023). Thermogravimetric kinetic-based computation of raw and pretreated coconut husk powder lignocellulosic composition. Bioresour. Technol. Rep. 22, 101500. 10.1016/j.biteb.2023.101500

[B12] Filippi S. Cinelli P. Mezzetta A. Carlozzi P. Seggiani M. (2021). Extraction of polyhydroxyalkanoates from purple non-sulfur bacteria by non-chlorinated solvents. Polym. (Basel) 13, 4163. 10.3390/polym13234163 PMC8659763 34883666

[B13] Gumel A. M. Annuar M. S. M. Heidelberg T. (2014). Growth kinetics, effect of carbon substrate in biosynthesis of mcl-PHA by Pseudomonas putida Bet001. Braz. J. Microbiol. 45, 427–438. 10.1590/S1517-83822014000200009 25242925 PMC4166266

[B14] Hierro-iglesias C. Fatokun C. O. Chimphango A. Bayitse R. Helena P. Thornley P. (2023). u rn a l P. J. Environ. Chem. Eng. 111815. 10.1016/j.jece.2023.111815

[B15] Jiang G. Hill D. J. Kowalczuk M. Johnston B. Adamus G. Irorere V. (2016). Carbon sources for polyhydroxyalkanoates and an integrated biorefinery. Int. J. Mol. Sci. 17, 1157. 10.3390/ijms17071157 27447619 PMC4964529

[B16] Kacanski M. Stelzer F. Walsh M. Kenny S. O’Connor K. Neureiter M. (2023). Pilot-scale production of mcl-PHA by Pseudomonas citronellolis using acetic acid as the sole carbon source. N. Biotechnol. 78, 68–75. 10.1016/j.nbt.2023.10.003 37827242

[B17] Kalafat E. Laoreti A. Khalil A. Costa F. D. S. Thilaganathan B. (2018). Ophthalmic artery Doppler for prediction of pre-eclampsia: systematic review and meta-analysis. J. Fish. Biol. 1, 731–737. 10.1002/uog.19002 29330892

[B46] Kenny S. Nikodinovic-Runic J. Kaminsky W. Woods T. Babu R. Keely C. (2008). Up-Cycling of PET (Polyethylene Terephthalate) to the Biodegradable Plastic PHA (Polyhydroxyalkanoate). Environ. Sci. Technol. 42, 7696–7701. 10.1021/es801010e 18983095

[B18] Khanal S. Karimi K. Majumdar S. Kumar V. Verma R. Bhatia S. K. (2023). Sustainable utilization and valorization of potato waste: state of the art, challenges, and perspectives. Biomass Convers. Biorefinery. 10.1007/s13399-023-04521-1

[B19] Lafont-Mendoza J. J. Severiche-Sierra C. A. Jaimes-Morales J. (2018). Evaluation of the starch quantification methods of *Musa paradisiaca, Manihot esculenta,* and *Dioscorea trífida* using factorial experiments. Int. J. Food Sci. 2018, 1–7. 10.1155/2018/5901930 PMC6258095 30538999

[B20] Liang S. McDonald A. G. (2014). Chemical and thermal characterization of potato peel waste and its fermentation residue as potential resources for biofuel and bioproducts production. J. Agric. Food Chem. 62, 8421–8429. 10.1021/jf5019406 25093245

[B21] Liang S. Han Y. Wei L. McDonald A. G. (2015). Production and characterization of bio-oil and bio-char from pyrolysis of potato peel wastes. Biomass Convers. Biorefinery 5, 237–246. 10.1007/s13399-014-0130-x

[B22] Liang S. McDonald A. G. Coats E. R. (2014). Lactic acid production from potato peel waste by anaerobic sequencing batch fermentation using undefined mixed culture. Waste Manag. 45, 51–56. 10.1016/j.wasman.2015.02.004 25708409

[B23] Lima M. de A. Andreou R. Charalampopoulos D. Chatzifragkou A. (2021). Supercritical carbon dioxide extraction of phenolic compounds from potato (Solanum tuberosum) peels. Appl. Sci. 11, 3410. 10.3390/app11083410

[B24] Mahato R. P. Kumar S. Singh P. (2021). Optimization of growth conditions to produce sustainable polyhydroxyalkanoate bioplastic by *Pseudomonas aeruginosa* EO1. Front. Microbiol. 12, 711588. 10.3389/fmicb.2021.711588 34721317 PMC8555948

[B25] Maheshwari N. Kumar M. Thakur I. S. Srivastava S. (2018). Production, process optimization and molecular characterization of polyhydroxyalkanoate (PHA) by CO2 sequestering B. cereus SS105. Bioresour. Technol. 254, 75–82. 10.1016/j.biortech.2018.01.002 29413942

[B26] Malakar B. Das D. Mohanty K. (2020). Optimization of glucose yield from potato and sweet lime peel waste through different pre-treatment techniques along with enzyme assisted hydrolysis towards liquid biofuel. Renew. Energy 145, 2723–2732. 10.1016/j.renene.2019.08.037

[B27] Osman A. I. Blewitt J. Abu-Dahrieh J. K. Farrell C. Al-muhtaseb A. H. Harrison J. (2019). Production and characterisation of activated carbon and carbon nanotubes from potato peel waste and their application in heavy metal removal. Environ. Sci. Pollut. Res. 26, 37228–37241. 10.1007/s11356-019-06594-w PMC6937222 31745803

[B28] Pan L. Li J. Wang R. Wang Yu Lin Q. Li C. (2021). Biosynthesis of polyhydroxyalkanoate from food waste oil by *Pseudomonas alcaligenes* with simultaneous energy recovery from fermentation wastewater. Waste Manag. 131, 268–276. 10.1016/j.wasman.2021.06.008 34175751

[B29] Passanha P. Kedia G. Dinsdale R. M. Guwy A. J. Esteves S. R. (2014). The use of NaCl addition for the improvement of polyhydroxyalkanoate production by Cupriavidus necator. Bioresour. Technol. 163, 287–294. 10.1016/j.biortech.2014.04.068 24835740

[B30] Remedios Y. Domingues L. (2023). Potato peels waste as a sustainable source for biotechnological production of biofuels. Process Optim. 155, 320–328. 10.1016/j.wasman.2022.11.007 36413884

[B31] Rodríguez-Martínez B. Coelho E. Gullón B. Yáñez R. Domingues L. (2023). Potato peels waste as a sustainable source for biotechnological production of biofuels: process optimization. Waste Manag. 155, 320–328. 10.1016/j.wasman.2022.11.007 36413884

[B32] Samarin A. M. Hematyar N. Elhamirad A. H. (2012). Phenolics in potato peels: extraction and utilization as natural antioxidants phenolics in potato peels: extraction and utilization as natural antioxidants. World Appl. Sci. J. 10.5829/idosi.wasj.2012.18.02.1057

[B33] Sampaio S. L. Petropoulos S. A. Alexopoulos A. Heleno S. A. Santos-buelga C. Barros L. (2020). Potato peels as sources of functional compounds for the food industry: a review. Trends Food Sci. Technol. 103, 118–129. 10.1016/j.tifs.2020.07.015

[B34] Shangdiar S. Cheng P. Chen S. Amesho K. T. Ponnusamy V. K. Lin Y. C. (2023). Enhancing sugar yield for bioconversion of rice straw: optimization of Microwave-assisted Pretreatment using dilute acid hydrolysis. Environ. Technol. Innov. 32, 103313. 10.1016/j.eti.2023.103313

[B35] Sharma P. K. Fu J. Cicek N. Sparling R. Levin D. B. (2012). Kinetics of medium-chain-length polyhydroxyalkanoate production by a novel isolate of pseudomonas putida LS46. Can. J. Microbiol. 58, 982–989. 10.1139/W2012-074 22804681

[B36] Sharma P. K. Munir R. I. Blunt W. Dartiailh C. Cheng J. Charles T. C. (2017). Synthesis and physical properties of polyhydroxyalkanoate polymers with different monomer compositions by recombinant Pseudomonas putida LS46 expressing a novel PHA SYNTHASE (PhaC116) enzyme. Appl. Sci. 7, 242. 10.3390/app7030242

[B37] Sikkema W. D. Cal A. J. Hathwaik U. I. Orts W. J. Id C. C. L. (2023). Polyhydroxyalkanoate production in Pseudomonas putida from alkanoic acids of varying lengths. Plos One 15, e0284377. 10.1371/journal.pone.0284377 PMC10358918 37471433

[B38] Soni S. K. Sharma B. Sharma A. Thakur B. Soni R. (2023). Potato peels to bioethanol: evaluation of different pre-treatment strategies for low-cost saccharification using in-house produced multi-enzyme system. Biol. Biotechnol. 19, 1. 10.20944/preprints202305.0635.v1

[B39] Sriyapai T. Chuarung T. Kimbara K. Samosorn S. Sriyapai P. (2022). Production and optimization of polyhydroxyalkanoates (PHAs) from Paraburkholderia sp. PFN 29 under submerged fermentation. Electron. J. Biotechnol. 56, 1–11. 10.1016/j.ejbt.2021.12.003

[B40] Tanikkul P. Sullivan G. L. Sarp S. Pisutpaisal N. (2020a). Biosynthesis of medium chain length polyhydroxyalkanoates (mcl-PHAs) from palm oil. Case Stud. Chem. Environ. Eng. 2, 100045. 10.1016/j.cscee.2020.100045

[B41] Tanikkul P. Sullivan G. L. Sarp S. Pisutpaisal N. (2020b). Biosynthesis of medium chain length polyhydroxyalkanoates (mcl-PHAs) from palm oil. Case Stud. Chem. Environ. Eng. 2, 100045. 10.1016/j.cscee.2020.100045

[B42] Vu D. H. Wainaina S. Taherzadeh M. J. Åkesson D. Ferreira J. A. (2021). Production of polyhydroxyalkanoates (PHAs) by Bacillus megaterium using food waste acidogenic fermentation-derived volatile fatty acids. Bioengineered 12, 2480–2498. 10.1080/21655979.2021.1935524 34115556 PMC8806590

[B43] Wang J. Liu S. Huang J. Cui R. Xu Y. Song Z. (2023). Genetic engineering strategies for sustainable polyhydroxyalkanoate (PHA) production from carbon-rich wastes. Environ. Technol. Innov. 30, 103069. 10.1016/j.eti.2023.103069

[B47] Xu Z. Pan C. Li X. Hao N. Zhang T. Gaffrey M. J. (2021). Enhancement of polyhydroxyalkanoate production by co-feeding lignin derivatives with glycerol in Pseudomonas putida KT2440. Biotechnol. Biofuels 14, 11. 10.1186/s13068-020-01861-2 33413621 PMC7792162

[B44] Zhou C. Jiang W. Via B. K. Fasina O. Han G. (2015). Prediction of mixed hardwood lignin and carbohydrate content using ATR-FTIR and FT-NIR. Carbohydr. Polym. 121, 336–341. 10.1016/j.carbpol.2014.11.062 25659707

[B45] Zhou W. Bergsma S. Colpa D. I. Euverink G. J. W. Krooneman J. (2023). Polyhydroxyalkanoates (PHAs) synthesis and degradation by microbes and applications towards a circular economy. J. Environ. Manage. 341, 118033. 10.1016/j.jenvman.2023.118033 37156023

